# 

**Published:** 1924-03

**Authors:** 


New Series. Vol. III. No. 30 (Vol. LXXIII. No. 1872). March, 1924. Monthly, Price 6d. (POST FREE 7½d.)

